# Regulatory Roles of the N-Terminal Intrinsically Disordered Region of Modular Src

**DOI:** 10.3390/ijms23042241

**Published:** 2022-02-17

**Authors:** Goro Kato

**Affiliations:** Laboratory of Biological Chemistry, Center for Medical Education and Sciences, University of Yamanashi, 1110 Shimokato, Chuo 409-3898, Yamanashi, Japan; gkato@yamanashi.ac.jp

**Keywords:** Src, intrinsically disordered region (IDR), SH4 domain, unique domain (UD), SH3 domain, phosphorylation, fuzzy intramolecular complex (FIMC), kinase domain, canonical model, nuclear magnetic resonance (NMR)

## Abstract

Src, the prototype of Src family kinases (SFKs), is a modular protein consisting of SH4 (SH4) and unique (UD) domains in an N-terminal intrinsically disordered region (IDR), and SH3, SH2, and kinase (KD) folded domains conserved among SFKs. Src functions as a pleiotropic signaling hub in proliferating and post-mitotic cells, and it is related to cancer and neurological diseases. However, its regulatory mechanism is unclear because the existing canonical model is derived from crystallographic analyses of folded constructs lacking the IDR. This work reviews nuclear magnetic resonance analyses of partially structured lipid-binding segments in the flexible UD and the fuzzy intramolecular complex (FIMC) comprising IDR and SH3 domains, which interacts with lipid membranes and proteins. Furthermore, recently determined IDR-related Src characteristics are discussed, including dimerization, SH4/KD intramolecular fastener bundling of folded domains, and the sorting of adhesive structures. Finally, the modulatory roles of IDR phosphorylation in Src activities involving the FIMC are explored. The new regulatory roles of IDRs are integrated with the canonical model to elucidate the functions of full-length Src. This review presents new aspects of Src regulation, and provides a future direction for studies on the structure and function of Src, and their implications for pathological processes.

## 1. Introduction

**Figure 1:**
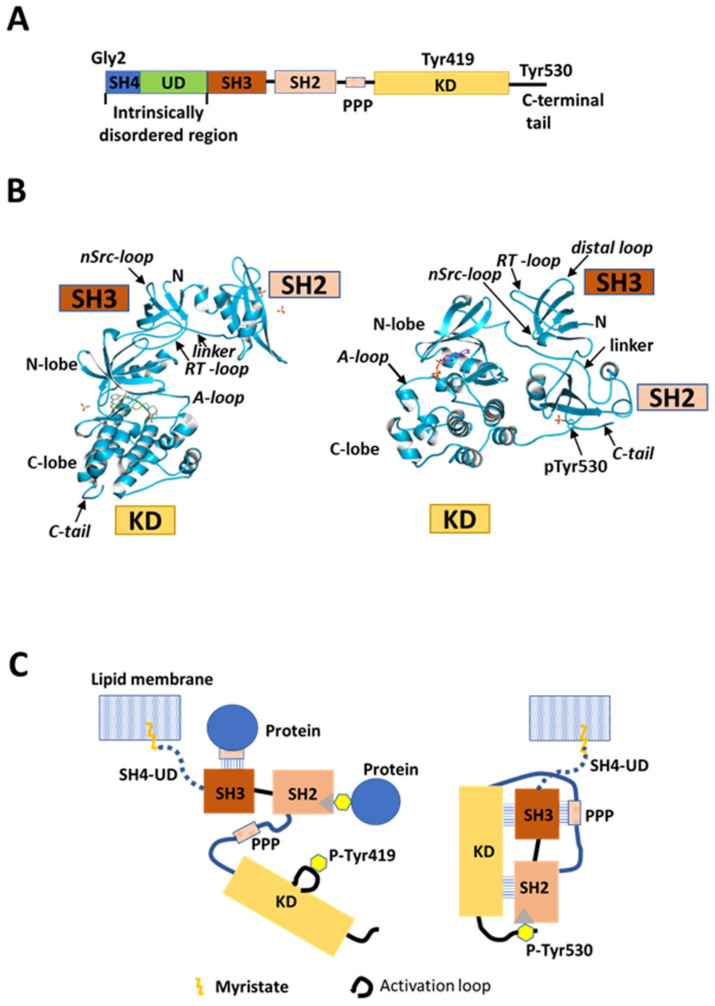
Src structure and regulation. (**A**) Schematic of domain structure of human Src. Five domains, SH4 (dark blue), unique domain (UD; green), SH3 (dark orange), SH2 (light orange), and kinase domain (KD; gold), are arranged sequentially from the N-terminus. SH2 and KD are connected by the SH2-kinase linker containing a poly-proline peptide (PPP). The N-terminal SH4, UD, SH3, and SH2 domains are collectively called the regulatory domain. SH4 and UD are positioned in an intrinsically disordered region (IDR), while the other domains are folded. KD binds substrate peptides and ATP to exert catalytic activity. Gly2 is a myristoylation site; Tyr419 is an autophosphorylation site in the activation loop in KD; Tyr530 is a phosphorylation site in the C-terminal regulatory tail. (**B**) Crystal structure (© PDBj) of closed (right; PDB: 2SRC [17]) and open (left; PDB: 1Y57 [20]) conformational states of Src SH3-SH2-KD construct lacking SH4 and UD. A-loop: activation loop; linker: poly-proline linker; pTyr530: phosphorylated tyrosine 530; N: N-terminus; C-tail: C-terminal tail. (**C**) Schematic representation of canonical model of Src kinase regulation. This model is based on X-ray crystallography analysis of the truncated construct in (**B**). Myristoylated SH4 is inserted into the lipid membrane, and UD is considered to be a simple rope linking the truncated construct to the membrane. Right: closed repressed form. SH3 interacts with the PPP segment in the SH2-kinase linker. SH2 interacts with phosphotyrosine 530 (P-Tyr530) in the C-terminal regulatory tail. Both SH3 and SH2 domains interact with KD. The activation loop adopts an inactive state in these repressed and closed truncated forms of Src. Left: open activated form. Dephosphorylation of phosphorylated Tyr530, and extramolecular interactions of phosphotyrosine-containing, SH2-binding, and PPP-containing SH3-binding proteins with Src SH2 and SH3 break the intramolecular interactions and open the closed conformation between SH3-SH2 and KD, resulting in an activated conformation of the activation loop autophosphorylated at Tyr419.

Src is a 60 kDa non-receptor protein tyrosine kinase and a mediator of pleiotropic molecular signaling [1,2]. Src is expressed ubiquitously in mammalian tissues and is involved in the regulation of cell growth and post-mitotic cell behavior [3,4]. This modular protein comprises five functional domains that are shared with the other Src family kinases (SFKs), consisting of Src-homology 4 (SH4), unique (UD), Src-homology 3 (SH3), and Src-homology 2 (SH2) domains, a poly-proline linker, a kinase domain (KD), and a C-terminal regulatory region, from the N-terminus to the C-terminus (Figure 1A) [5]. SFKs share high sequence homology, except for the N-terminal region comprising SH4 and UD components. This N-terminal segment shares minimal primary sequence homology among SFK family members, and the only conserved amino acids in the SH4 domain are Gly2 and Cys3 (except Src). These two conserved residues are sites for myristoylation and palmitoylation, respectively, and contribute to membrane anchoring [6,7,8,9] and subcellular localization and transport [10,11]. Positively charged residues in SH4 of Src also contribute to interactions with negatively charged membrane phospholipids [12,13]. Regulation of the catalytic activity of Src has been proposed based on X-ray crystallographic studies on conserved SH3, SH2, and KD components (Figure 1B,C). SH3 and SH2 are regulatory domains [5]. Interaction of SH2 with a phosphorylated tyrosine (Y530 in human Src) located in the C-terminal region [14] contributes to maintaining Src in a basal closed state that is enzymatically inactive [15] (Figure 1B, right, and Figure 1C, right). Another type of regulation has been associated with interactions involving SH3 and SH2; SH3 binds to a poly-proline-peptide (PPP) in the linker connecting SH2 and KD [16,17], and there are interactions between SH3 and the N-lobe of KD involving nSrc and RT loops [18]. Furthermore, recent large-scale mutagenesis/crystallography analyses showed that SH2 interacts with the C-lobe of KD to stabilize the closed state [19]. The enzymatically active open conformation of the bilobal KD is adopted by releasing these intramolecular interactions, and this promotes Y419 autophosphorylation and extramolecular interactions of the regulatory domains, the poly-proline linker, and the tyrosine-phosphorylated C-terminal region (Figure 1B, left, and Figure 1C, left) [20].

**Figure 2:**
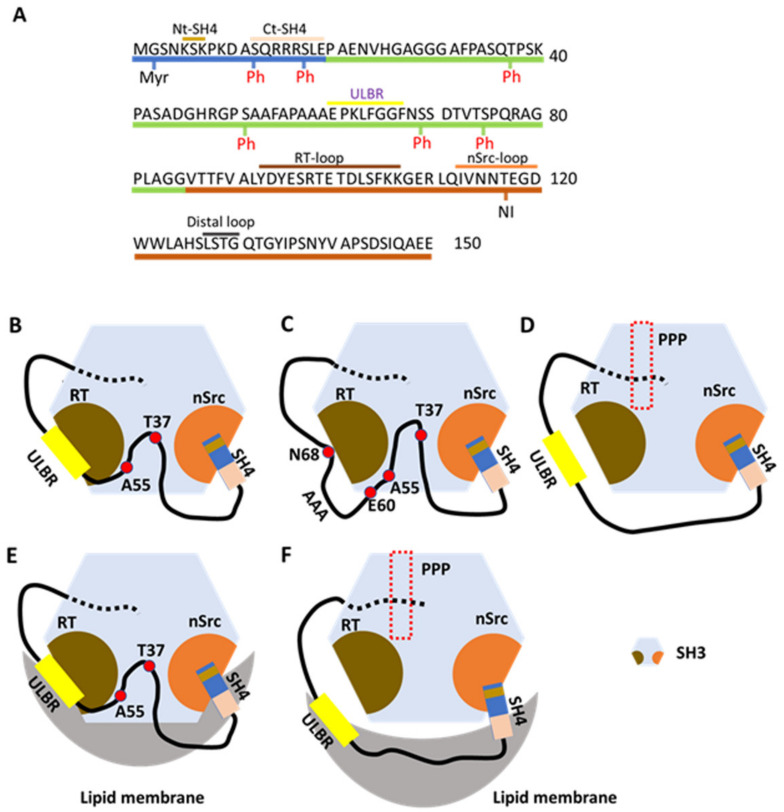
The fuzzy intramolecular complex (FIMC) of N-terminally intrinsically disordered non-myristoylated USrcSH3 scaffolded by SH3. (**A**) The sequence of residues in IDR (SH4 and UD) and SH3. Residues 1–19 are contained in SH4; residues 20–85 are contained in UD; and residues 86–150 are contained in SH3, which are colored as in Figure 1A. Nt-SH4 and Ct-SH4 contain residues 5–7 and 12–19. ULBR, a partially structured region in UD, contains residues 60–67. Three loops, RT, nSrc, and distal loops between each β-sheet (not designated) in SH3, are labeled above the residues. Myristoylation (Myr) and phosphorylation (Ph) sites are shown beneath the residues. Neuronal insertion (NI) occurring in N-Src is also shown under the residues. (**B**) UD interacts with the RT loop of SH3 through residues Thr37, Ala55, and ULBR (between E60 and N68). SH4 interacts with the nSrc loop of SH3 through its N-terminal residues (Nt-SH4). (**C**) Effects of ULBR mutation. The 3A mutant of UD, in which residues LFG in ULBR are replaced by three alanines, displays a small decrease in interaction with SH3, but retains the overall interaction. The 3A mutation disrupts the partially structured state. (**D**) In the presence of PPP (derived from the poly-proline peptide in the linker between SH2 and KD). PPP binding allosterically inhibits the interaction between UD and SH3, but not the interaction between SH4 and SH3. (**E**) In the presence of lipid membranes. SH3 binds to lipid membranes through its RT and nSrc loops. The ULBR binds to lipid membranes and SH4 interacts with lipid membranes through its C-terminal residues (Ct-SH4). Interaction between the SH4-UD and SH3 is retained. (**F**) In the presence of lipid membranes and PPP. PPP abolishes the interaction between SH3 and lipid membranes, as well as the interaction between UD and SH3. SH4-related interactions with SH3 and lipid membranes are retained. ULBR binding to lipid membranes is also retained. Figure 2B–F is adapted from Figure 8 in a previous report [34] (p. 901).

Both SH4 and UD of Src are intrinsically disordered domains (Figure 1A and Figure 2A), and intrinsically disordered proteins (IDPs) or proteins with intrinsically disordered regions (IDRs) typically undergo dynamic intermolecular and intramolecular interactions through the formation of multivalent complexes and fuzzy complexes [21,22]. IDPs contribute to many biological functions including the regulation of signal transduction pathways [23,24]. IDPs have been linked to cancer, neurodegeneration, and other diseases [25,26,27,28]. The IDR of Src consists of SH4 and UD, and it is related to membrane attachment [13,29], protein–protein interactions [30], and neuronal survival [31]. However, the full-length structure of Src containing the N-terminal SH4 and UD regions cannot be analyzed by crystallography, and the regulatory function cannot be fully understood from crystal structures, but the N-terminal region of each individual SKF member is well conserved between different organisms, implying important and specific regulatory roles [32].

However, nuclear magnetic resonance (NMR) studies on the structure and function of the unfolded regions of Src have revealed the IDR-associated regulation of Src activities [21,33,34,35,36], providing insight into overall regulation and canonical Src kinase regulation. Furthermore, SH4-UD in the IDR regulates dimerization/oligomerization of Src proteins [37] and the modulation of membrane anchoring via myristoylated SH4, which interacts with SH3 domains, as revealed by NMR studies on the IDR of Src [38]. Furthermore, optogenetic research and chemical genetic methods combined with comprehensive mutagenesis has revealed novel aspects of Src molecular behavior and activity controlled by the IDR [19,39].

IDRs are sites of protein phosphorylation that facilitate the rapid regulation of protein function [40]. Electrostatic interactions involving phosphorylation may affect conformationally flexible IDR-containing proteins, enabling the modulation of protein–protein interactions and signaling [41]. The UD region of Src contains several sites that undergo phosphorylation and dephosphorylation in response to diverse cellular processes (Figure 2A), and phosphorylation sites of other members of SFKs are different from those of Src [32]. The structure and function of the IDR in Src and the biological and biochemical relevance of modulation by phosphorylation/dephosphorylation have been investigated by in vitro and in vivo real-time NMR studies [33,42], combined with molecular and cellular biological approaches [30,43,44,45,46].

The structures and functions of IDRs of SFKs are poorly understood, despite focused investigations on individual folded domains. Src is the prototype of the SFK family, and the IDR of Src has received considerable attention from a structure and function perspective. This review discusses new aspects of the structure and function of Src based on NMR analysis of its IDR. Furthermore, studies on new regulatory roles of the IDR of Src are discussed, including regulatory phosphorylation events.

## 2. NMR Structural Characterization of Src IDR

### 2.1. Lipid Binding by Src UD through the Unique Lipid-Binding Region (ULBR)

NMR is well suited for the study of proteins containing IDRs. Perez et al. [35] first characterized USrc (SH4 and UD of human Src kinase, residues 1–85) using a G2A non-myristoylated mutant. Two NMR parameters, residual dipolar coupling (RDC) and paramagnetic relaxation enhancement (PRE), were employed to characterize conformational plasticity and long-range interactions between remote regions of the IDR. They revealed the presence of transient secondary structural elements encompassing a partially helical structured segment spanning residues 60–65 and 67–75. Long-range contacts were not evident in USrc.

USrc, a non-myristoylated construct, was studied in the presence of model membranes consisting of a small disk-like structure formed by planer bilayers of long-chain lipids enclosed by micelle-like walls made from short-chain lipids [36]. Chemical shifts on NMR spectra in the presence of lipids were observed for SH4 and UD residues S51, A53, and A55, and residues 60–67 (EPKLFGGF). The observed shifts in UD were larger than those observed for the well-known lipid-binding SH4 domain. Residues 60–67 are within the partially structured region (residues 60–75) described above, referred to as the ULBR (Figure 2A) [36]. Replacing residues 63–65 (LFG) by three alanines had only a minor effect on lipid binding based on NMR chemical shifts in USrc AAA [36]. Replacing residues 63–68 (LFGGFN) by AAAEAE (EAE mutant) caused no lipid-induced NMR chemical shift changes in USrc EAE.

The Src construct USrc binds preferentially to acidic lipids including phosphatidylinositols according to lipid strip tests, and chemical shift perturbations (CSPs) in the ULBR were observed in the presence of neutral lipid bicelles, in contrast to residues in SH4 [36]. Identifying targets or niches of the ULBR on the plasma membrane and intracellular membrane in living cells could help to reveal Src behavior in membrane trafficking.

### 2.2. Interaction of USrc with the SH3 Domain

NMR CSPs between isolated and SH3-linked domains of USrc (USrcSH3, residues 1–150; i.e., between USrc vs. USrcSH3 and SH3 vs. USrcSH3) identified interacting residues in the nSrc loop, the RT loop, and the distal loop of SH3 (Figure 2A) [34,36]. SH3 interacts with UD through its RT loop and with the SH4 domain through its nSrc loop (Figure 2B). NMR CSPs showed that SH3 interacts with UD through residues T37 and A55, and ULBR residues E60 and K62 in UD; SH3 also interacts with SH4 through residues 5–7 in SH4 (Nt-SH4 in Figure 2A,B).

Residues 63–65 (LFG) were replaced by three alanines (3A mutant), and the construct was used to analyze the effects of UD ULBR on interactions between USrc and SH3. The AAA UD mutant and wild-type (WT) UD displayed similar CSPs for SH3 residues, although small reductions were observed in the AAA mutant in the distal loop and the RT loop. Thus, UD harboring the AAA mutation is still able to interact with SH3 (Figure 2C). Comparison of PRE effects in isolated WT and AAA forms of USrc showed that the AAA expands the random-coil structure, which may relate to the phenotypes observed for the full-length Src AAA mutant described below [36].

The SH3 region interacts with USrc containing RT and nSrc loops located opposite the PPP-binding site, related to the canonical repressed conformation and extramolecular interactions [5]. Interestingly, binding of PPP to SH3 allosterically inhibits its interaction with UD (Figure 2D) but not with SH4 [34,36]. Interactions between many UD residues and SH3 are disrupted by the presence of PPP. In summary, Nt-SH4 interacts with the nSrc loop of SH3 in the absence or presence of PPP, whereas the interaction between the RT loop and UD is lost in the presence of PPP (Figure 2B,D).

Using a synthetic peptide harboring the SH4 sequence (residues 2–19), the interaction between SH4 and SH3 was analyzed, revealing that the addition of SH4 peptide to PPP-SH3 and SH3 results in small and similar CSPs in the RT loop, the nSrc loop, and the distal loop [34]. By contrast, large SH4 peptide-induced CSPs in the nSrc loop were observed in USrcSH3 (SH4 and SH3 are connected by UD) in the presence and absence of PPP. Thus, UD facilitates interactions between SH4 and the nSrc loop in the presence and absence of PPP. SH3 acts as a scaffold for the intrinsically disordered UD, concomitant with the interaction with SH4, which bundles a long loop including the whole UD region [34]. Additional contacts between residues in UD and SH3 further decrease its conformational freedom. Contacts with UD are lost in the PPP complex, but a flexible loop is retained (Figure 2D).

### 2.3. Lipid Binding by USrc Attached to SH3

Although lipid-induced chemical shifts were very similar in isolated USrc or USrcSH3, shifts were evident for residues 98–102 (RT loop) and 114–116 (nSrc loop) of SH3, the opposite side of the canonical PPP binding site, both in isolated SH3 (residues 86–150) and USrcSH3 [34,36], indicating that human Src has two lipid-binding regions (RT and nSrc loops of SH3), in addition to SH4 (Figure 2E). Isolated SH3 showed lipid-binding selectivity similar to that of isolated USrc. However, USrcSH3 did not bind phosphoinositides, and the selectivity differed from that of isolated SH3, suggesting that the two domains interact [33,36].

Direct lipid binding of SH3 was almost completely inhibited by PPP binding (Figure 2F), but lipid interaction between ULBR residues 64–67 and Ct-SH4 residues 14–17 (Figure 2A) was retained following PPP binding (Figure 2F), as revealed by comparing the chemical shifts of PPP-bound and unbound constructs of USrcSH3 [34]. However, in the presence of PPP, the chemical shifts of ULBR residues of USrcSH3 do not accord with those of USrc in the presence of lipids, suggesting that lipid binding by ULBR is retained but affected by the proximity of the SH3 domain. On the other hand, in the absence of PPP, T37 and A55 and ULBR residues in UD interact with SH3 in the presence of lipids (Figure 2E). During PPP binding, interactions between UD and SH3 are also abolished in the presence of lipids (Figure 2F) [34].

SH4 is the primary site anchoring Src to membranes, but SH4 also interacts with SH3. Furthermore, SH4 simultaneously interacts with lipids and SH3 [34]. Residues in Nt-SH4 bind SH3, and this binding is marginally affected by lipids in USrcSH3 in the presence or absence of PPP (Figure 2B,E,F). On the other hand, residues in Ct-SH4 exhibit the largest CSPs following lipid binding, and interaction with Ct-SH4 residues is retained in USrcSH3 bound to PPP (Figure 2E,F).

### 2.4. Neuronal Form of Src

Neuronal Src (N-Src) has a 6 or 17 amino acid insert in the nSrc loop of SH3 (Figure 2A) depending on alternative splicing between exons 3 and 4 [47,48,49]. N-Src has activated phosphotransferase activity in differentiated central nervous system (CNS) neurons [50,51]. Fyn and Yes, highly homologous to Src, do not contain neuron-specific alternative exons, although they exhibit higher kinase activity [49]. The molecular and structural basis of the activation has been revealed based on the canonical interaction of the SH3-SH2-kinase linker with KD, which represses Src kinase activity, as well as by the characterization and identification of a neuronal SH3-interacting protein (Src substrate) [52,53,54]. Transgenic mice expressing constitutively active N-Src in the cerebellum show the aberrant morphology of Purkinje dendrites in the development [55]. However, the mechanisms of the neuronal activation and physiological function of N-Src are unclear. To gain greater insight into the roles of Src in post-mitotic neurons, future studies will need to explore the effects of the insertion of a neuronal peptide on the interactions of residues in IDR with SH3 in the presence and absence of lipids and PPP peptides.

## 3. The Fuzzy Intramolecular Complex (FIMC) Comprising IDR and Structured SH3 Domain Features

### 3.1. Scaffolding Role of SH3

Small-angle X-ray scattering, which monitors the degree of compactness, has confirmed the compact structure of USrcSH3 and highlighted the scaffolding role of SH3 in forming a FIMC consisting of IDR of SH4 and UD, and SH3 in USrcSH3 [33]. PRE NMR experiments (∆PRE; deviations between experimental PRE and predictions from a random coil model) on USrcSH3 and USrc show that most long-range interactions in the IDR of USrcSH3 can be retained without SH3, indicating that the IDR is pre-organized in the absence of SH3. The pre-organized structure may restrain the flexible peptide chain in the proximity of SH3 [33]. The newly observed interaction between SH4 and UD, between residues 15–16 and the ULBR (residues 65–66) only in the presence of SH3, indicates its scaffolding role [33].

### 3.2. Conserved Sequence Features in UD and a Novel Src Regulation System

Although the sequences of UD among SFK members share very low similarity, the interacting SH3 is highly conserved, suggesting that common sequence features likely promote functional interactions with SH3 [33,56]. Aromatic resides (positions 32 and 54 in human Src) are highly conserved, and residues between these conserved amino acids include three to four proline residues in Src, Fyn, and Fgr. Proline residues might save the entropic cost of compacting the disordered SH4 and UD [33]. Another conserved feature is found in the Φ1xxΦ2 pattern of ULBR, in which Φ1 is a phenylalanine or tyrosine, x is a turn-promoting residue (G, S, N), and Φ2 is an aromatic or hydrophobic residue [33].

Mutation of the phenylalanine residues of UD cause the loss of long-range interactions based on CSPs in USrcSH3, confirming long-range interactions of the aromatic residues within the disordered region [33]. F32A and F54A mutations induced shifts in the same SH3 regions (i.e., distal and nSrc loops and β-strands). By contrast, F64A and F67A mutations in ULBR caused CSPs mainly in the RT loop. A direct interaction between the ULBR and the RT loop, in relation to variations in these regions, has been reported for viral Src [18,57]. The combination of proline and aromatic residues seems to favor a compact state in Src UD, which is pre-organized to allow multiple weak contacts with SH3 [33].

IDRs of SFKs have common (conserved) strategies in the formation of the FIMC of IDR and SH3. However, they have specific sequences responsive to specific inputs. Each SFK receives a specific signal through a specific UD to trigger a specific output through a shared common mechanism integrating disordered domains and structured SH3 domains into FIMCs [21,34,56].

### 3.3. Biological Effects of the IDR of Full-Length Src: In Vivo Effects of Mutations in the ULBR

Whether or not the compact FIMC in USrcSH3 plays physiological roles in the context of the entire Src protein is interesting, and it has been investigated using two systems.

In the Xenopus oocyte system, progesterone-induced maturation is promoted by active viral Src and Xenopus Src. Oocytes were injected with in vitro-transcribed mRNA encoding constitutively active human Src or SrcY530F, with either WT, AAA, or EAE mutants of the ULBR of UD [36]. Treatment with the WT protein caused oocytes to begin maturation, accompanied by germinal vesicle breakdown and meiosis I entry approximately 2 h before control oocytes. AAA or EAE mutants similarly triggered maturation in response to progesterone, but maturation events were only 70–80% complete. At 2 h after the appearance of the signs, about half of the mutants exhibited progressive depigmentation and started to die. Thus, ULBR plays an essential role in the regulation of Src activity.

In the human cancer cell system, SW620 cells isolated from a metastatic ganglion of a colorectal carcinoma expressed low levels of endogenous SFK and exhibited moderate invasive activity. Because cancer cells overexpressing WT full-length Src displayed increased cell invasiveness, cell invasion was compared between WT Src and the Src AAA mutant [33]. AAA mutant cells displayed decreased invasion ability by >50% with respect to WT cells, highlighting the critical role of the ULBR in the IDR in the context of full-length myristoylated Src. Elucidating the underlying molecular signaling mechanism requires further investigation.

## 4. FIMC-Based Modulation of Lipid Membrane Anchoring by Interaction of a Myristoyl Group with SH3

Membrane binding is crucial for the transforming activity of viral Src and for the activation of Src by a membrane-bound protein tyrosyl phosphatase [58]. All SFKs are myristoylated at the N-terminus of these SH4 domains [9], which contributes to membrane anchoring. The molecular mechanism promoting trafficking between the plasma membrane and intracellular organelles is poorly understood [10]. Vesicular trafficking is involved in solubilizing proteins that recruit Src released from the membrane [59]. The membrane-unbound form accounts for ~30% of intracellular Src [60]; thus, whether or not the binding pocket of the myristoyl group in the non-bound form is present is an intriguing question.

### 4.1. The Myristoyl Group Interacts with SH3, and ULBR Contributes to Its Interaction

**Figure 3:**
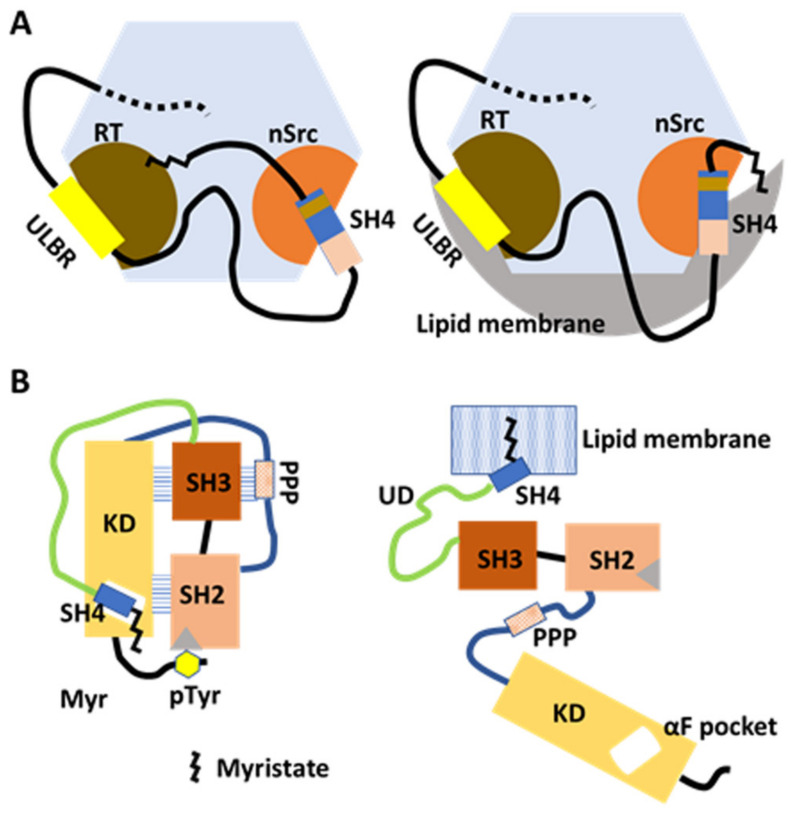
Membrane anchoring and intracellular transport modulated by myristate/SH3 interaction in the FIMC and by SH4/KD binding in the canonical model. (**A**) Model based on NMR structural analysis of myristoylated FIMC. The N-myristoyl group interacts with hydrophobic residues around the RT loop of SH3 in the absence of lipid membranes. The FIMC structure is maintained under myristoylation (left). In the presence of lipid membranes, the N-myristoyl group is detached from the SH3 and inserts into the membrane (right). UD contributes to the maintenance of the fuzzy complex in the absence and presence of lipid membranes. (**B**) Model based on comprehensive mutagenesis with chemical genetics. In the absence of lipids, SH4 binds the αF pocket, leading to the formation of the folded SH3/SH2/KD complex in the canonical model. Interaction between the N-terminal myristate and the αF-pocket in the C-lobe of KD is not confirmed (left). In the presence of lipid membranes, the N-myristoyl group binds to the lipid membrane to anchor the Src protein to the membrane (right).

NMR and surface plasmon resonance (SPR) studies have characterized the myristoylated N-terminal region construct (MyrUSrcSH3) in solution and its binding to liposomes [38]. CSPs from NMR spectra were obtained from two constructs (MyrUSrcSH3 and USrcSH3), and the same constructs were measured in two conditions (in the presence or absence of liposomes). Myristoylation-induced CSPs from MyrUSrcSH3 and USrcSH3, and CPSs for isolated SH3, showed that the N-myristoyl group binds to SH3 in the proximity of the RT loop, wherein a number of hydrophobic residues are located, in the absence of liposomes (Figure 3A, left). The FIMC is retained in the presence of the myristoyl group, since very few CPSs of key residues were affected by the interaction [38].

CSPs between MyrUSrcSH3AAA and USrcSH3AAA showed that the N-myristoyl group interacts with SH3 in the proximity of the RT loop, as observed for USrcSH3WT, but a distinct pattern of CSPs at some residues in the β1 strand and the RT loop of SH3 suggest that ULBR may affect the way that the N-myristoyl group binds to SH3 [38].

### 4.2. UD and SH3 Modulate Membrane Anchoring

In the presence of liposomes, myristoylation-Induced CSPs of RT loop residues from MyrUSrcSH3 and USrcS H3, with respect to the isolated SH3, are reminiscent of those of typical free SH3, suggesting that the myristoyl groupis released from the RT loop region of the SH3, accompanied by its insertion into lipid membranes (Figure 3A, right). Residues affected by interactions between SH3 and UD are not affected in MyrUSrcSH3 in the presence of liposomes, indicating that the FIMC of USrcSH3 is retained when the myristoyl group is released [38].

Comparing the effect of myristoylation of WT USrcSH3 with that of the USrcSH3AAA mutant in which ULBR is inactivated showed that the ULBR contributes to interaction of the myristoyl group with SH3, and FIMC is maintained when Src is bound to lipid membranes [38].

SPR analysis of reversible binding kinetics and the affinities toward liposome-immobilized chips were assessed for MyrUSrcSH3, isolated MyrSH4, MyrUSrcSH3AAA, and MyrUSrcSH3QAQ (residues 98–100, RTE in the RT loop replaced by QAQ, a pair of oppositely charged residues replaced by neutral glutamine). Both the AAA mutation in the UD ULBR and the QAQ mutation in the SH3 RT loop increased the lipid-binding affinity via faster association, although the dissociation rate was very similar, suggesting that UD and SH3 modulate the way that the liberated SH4 is anchored to the membrane [38]. UD and SH3 may contribute to the regulation of the transition between non-membrane-bound and membrane-bound forms of Src.

The direct observation of full-length myristoylated Src by NMR, in vitro or in vivo, will be needed to answer these questions, but technical barriers must be overcome [38] because the IDR of Src cannot be observed by X-ray crystallography analysis.

## 5. Intramolecular Interactions between Src SH4 and KD Revealed by Combined Saturation Mutagenesis and Chemical Genetics, and Their Relevance to Src Activity

### 5.1. An SH4 ‘Fastener’ Retains the Closed Intramolecular Complex of Regulatory Domains and KD

To evaluate the role of the N-terminal regions in the regulation of Src kinase activity, Ahler et al. [19] combined a chemical genetic method for controlling Src conformation with conformation-selective and ATP-competitive probes, with comprehensive mutagenesis of KD assessed by yeast growth. SH4 serves as a ‘fastener’, promoting the intramolecular interaction between the regulatory domains of SH2 and SH3, as well as KD, when SH4 interacts with the αF pocket in the C-terminal lobe of Src KD (Figure 3B). The SH4 fastener contributes to maintaining minimal phosphotransferase activity, restraining the accessibility of SH2 and SH3 to extracellular binding proteins, and modulating changes in phosphotransferase activity-independent cell morphology and membrane association. Physical interaction between SH4 and KD was confirmed by pulldown with an immobilized non-myristoylated SH4 versus WT Src KD, and with αF pocket mutants E381T and I444K (WT protein displayed efficient pulldown while mutants did not). How the SH4/αF pocket interaction affects autoinhibition by SH2/C-terminal phosphotyrosine interaction remains obscure [19].

### 5.2. Is the SH4 Fastener Model Consistent with the FIMC?

The FIMC, including SH4, UD, and SH3, involves a modulable interaction between SH4, UD, and SH3 [33,34]. However, how this compact structure involving contacts between SH4 and the SH3 nSrc loop is related, in a competitive or complementary manner to the SH4/KD fastener, has not been uncovered in the absence of the lipid membrane. The process of anchoring Src to the membrane might include an interchange between SH4/KD and SH4/SH3 binding (Figure 3). The flexible UD might be a key choreographer, but further structural studies with full-length Src are required to answer these questions.

## 6. Src Dimer Formation Is Mediated by Myristoylated UD

### 6.1. Involvement of the N-Terminal SH4-UD and KD in Src Dimer Formation

Src dimer formation has been suggested from gel filtration and sedimentation velocity analyses [61,62], but the molecular basis of dimerization and the structural and functional relevance between Src monomers and dimers remain unclear. To directly detect dimerization, all constructs, including full-length human Src and various deletion mutants lacking one or more of its functional domains with tags at the C-terminus, were expressed transiently in Src-Yes-Fyn null (SYF) cells [37]. The N-terminal myristoylated SH4-UD region and the KD were shown to be the functional determinants of dimerization. Neither the N-terminal SH4-UD nor the KD are able to dimerize with themselves [37].

Evidently, the N-terminal SH4-UD is only required on one partner for dimerization. However, the deletion of KD in either partner impairs dimerization [37], suggesting that dimerization is asymmetric.

### 6.2. Src Dimers in Living Cells Are Linked to Y419 Autophosphorylation and an Open Conformation

Dimerization is shown to be linked with the activation state and conformational dynamics, as demonstrated by dimerization assays using the Y530F mutant (trapped in the open state by destabilizing the Y530-SH2 interaction) and the 530YEEI mutant (trapped in the closed state by stabilizing the Y530-SH2 interaction). Y419 autophosphorylation is required for dimerization, based on Y530F mutation and K298M kinase-dead mutation analyses [37]. The addition of a Src kinase inhibitor shows that autophosphorylation is a prerequisite for dimerization, but it is thereafter dispensable once the dimer is formed [37].

**Figure 4:**
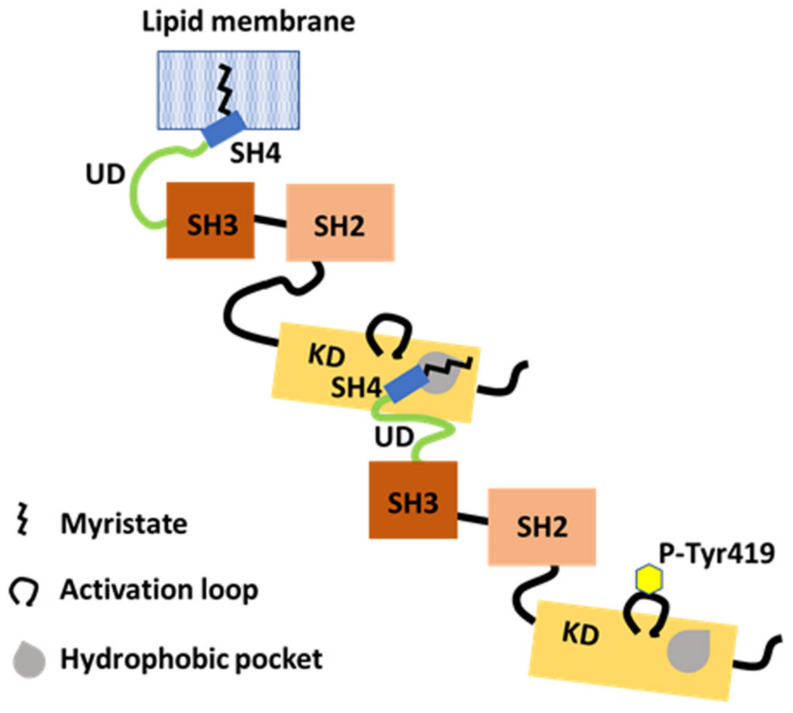
The N-terminal IDR supports dimerization. The myristoylated SH4-UD of one partner with Tyr419 autophosphorylation (P-Tyr419) interacts with the KD of another partner. The N-terminal myristate binds in a hydrophobic pocket in KD in trans. Dimerization and Tyr419 autophosphorylation are dependent on each other.

The N-terminal region of SH4 and UD, and autophosphorylation, are required in only one partner for dimerization, and both are required in cis (Figure 4) [37]. Furthermore, Y419 autophosphorylation was demonstrated to be an intermolecular event using the K298M kinase-dead mutant with a 20 kDa C-terminal SNAP tag in one partner [37]. Therefore, autophosphorylation is intermolecular and involves another partner, because Src cannot autophosphorylate Y419 intramolecularly [63,64].

### 6.3. Involvement of the N-Terminal Myristoyl Moiety in Binding a KD Pocket in Trans

Abl, a Src-related kinase, is myristoylated similarly to Src, but its myristoyl group can interact with the C-lobe of the KD [65,66]. It has been suggested that Src can bind to myristate at the KD site based on crystallographic and NMR studies on structural states of Src constructs lacking SH4 and UD [20].

The binding of recombinant purified SH4-UD to a purified KD indicates an interaction between the N-terminal region of Src and the C-terminal KD of Src [37]. Mutational research using the restrictive T459F and the permissive L494A mutations, introduced in trans with the N-terminal region, in the region of the putative hydrophobic pocket in the C-lobe of Src KD, indicate that dimerization is mediated by the myristoylated N-terminal region of one partner and by the hydrophobic pocket within the C-lobe of KD of the other partner (Figure 4) [37].

In the SH4 fastener model, SH4 binds to the αF pocket in the C-terminal lobe of Src KD, and the myristoyl group may contribute less to the binding [19]. By contrast, the N-terminal myristate of one partner binds to the hydrophobic pocket in the C-lobe of the KD, which overlaps with the αF pocket of another partner [37]. Following the intramolecular release of the SH4 fastener, the myristoylated SH4-UD of one partner might couple with a hydrophobic pocket in the C-lobe of the KD of another. However, how the UD segment contributes to specific binding to the C-lobe of KD during dimer formation is unknown at a structural level. Strict and detailed molecular analyses of the exchange between monomer and dimer/oligomer are needed in future work.

### 6.4. The Contribution of SH4-UD-Associated Dimerization to Kinase and Signaling Activities

Because the deletion of the N-terminal SH4-UD region reduces Src autophosphorylation, the region is not only important for dimerization; it also contributes to kinase activity [37]. The concentration–activity relationship for WT Src, revealed by in vitro autophosphorylation kinase assays, is consistent with an increase in the kinase-specific activity of Src as a function of concentration, which is consistent with dimerization-induced kinase activation. Moreover, the Y419F-K298M-Y530F triple mutant, possessing dominant negative activity through interference with Src dimerization, fails to self-dimerize. It does bind to WT Src, and it reduces the autophosphorylation activity of WT Src and the cellular phosphorylation activity of the substrate FAK [37]. Dimerization mediated by the N-terminal SH4-UD region enhances intracellular Src activity.

### 6.5. N-Terminal IDR-Assisted Dimerization and Y419 Phosphorylation Are Codependent Events, Revealing New Roles for Y419 Phosphorylation

Why phosphorylation of Y419 in cis is required for the N-terminal region to engage the KD of a partner in trans is an interesting question. The myristoyl group may interact with the same Src SH3 [38], or with the KD of its partner in the Src dimer [37]; thus, both interactions might be linked. Y419 phosphorylation in the KD, which alters the interaction between SH3 and KD, is required in cis (i.e., in the same molecule whose myristoyl group that binds to the SH3 is switched with that of the KD of the second Src involved in dimerization).

Reduced signaling and biological activities have been observed for the Src Y419F mutant, and evidence from other kinases shows that phosphorylation in the activation loop of their KD regions is essential for the active state to be adopted [67,68]. However, the closed and inactivated form of Src can be phosphorylated at Y419, and the two phosphorylation events at Y419 and Y530 are not mutually exclusive, suggesting that Src family members might activate each other, or be activated by other unknown kinases [69].

Y419 is phosphorylated in the closed and repressed conformation of Src [61]. Cowan-Jacob et al. [20] reported crystallographic studies on SH3, SH2, and Src KD unphosphorylated at Y419 and Y530, and the results indicated an extended activation loop with the active site exposed, consistent with the conformation of an active kinase (Figure 1B, left). Although Y419 phosphorylation alters the biological behavior of Src, this is not due to switching catalytic activity on or off. A functional relation between Y419 phosphorylation and dimerization could provide further insight into the role of Y419 phosphorylation [37]. Y419 phosphorylation may help Src to form specific molecular complexes through dimerization. In this way, it may affect access to substrates and selection by Src, serving more specific functions rather than simply increasing catalytic activity. Differences between monomers and dimers in the physiological functions of Src have been hypothesized, and transition between monomers and dimers might be dependent on Y419 phosphorylation [37].

### 6.6. Dimerization and Src Signaling Hub Activity

The stoichiometry of dimerization remains poorly understood. Future studies will need to more accurately determine the proportions of Src involved in monomers vs. dimers vs. larger oligomers, as well as address the dynamics of these macromolecular events [37].

Dimerization is unlikely to correspond to catalytic on or off switches, because although dimerization of the Y419F mutant of Src is impaired, it retains Src activity both in vitro [70,71] and in vivo [72,73]. Dimerization is more likely to affect the biological functions of Src. Because dimerization via the N-terminal region and KD mediates membrane localization, it may affect the regulation of Src activity and interactions with other proteins including kinases and phosphatases, which are mediated by the UD. Inherent flexibility in the UD in the FIMC may be crucial for its role as a regulatory hub that integrates all Src activities. It is possible that Src-Fyn heterodimers, and heterodimeric and homodimeric complexes of Src, may broaden the diversity of potential signaling activities, depending on the divergence of UDs among SFKs [37].

## 7. Biological Relevance of Src Dimerization/Oligomerization Is Dependent on UD and SH3: Insight from an Optogenetic Approach

### 7.1. Manipulating Src Signaling Using an Optogenetic Probe (optoSrc)

Kerjouan et al. [39] focused on the mechanism of pleiotropic signaling activity. To decipher the ability of Src to generate two different actin-associated structures (i.e., lamellipodia and invadosomes at adhesive sites on the plasma membrane), an optogenetic and photoactivable Src probe (optoSrc) based on a cryptochrome 2 (CRY2) optogenetic module was selected to activate and control Src spatiotemporally [39]. OptoSrc lacks an N-myristoyl membrane-anchoring domain and has a non-phosphotyrosine-binding R175L mutation in SH2 and an open conformation-inducing Y530F mutation. OptoSrc, which is not accumulated in any cellular compartment in the dark in Madin-Darby Canine Kidney (MDCK) cells, is directed to generate each adhesive structure by controlling the dynamics of optoSrc dimers/oligomers, which is induced by the ability of CRY2 to oligomerize, in adhesive sites formed by photostimulation [39].

The different decision-making events regulated by optoSrc dynamics induce the activation of various physiological substrates in Src signaling pathways. Relocalization of the optoSrc dimer to adhesive sites is promoted by SH3 to recognize the PPP of other proteins and to control Src kinase activity [39]. Src dimerization/oligomerization may lead to phase separation induced by SH3 domain multimers [56,74], which might favor SH3 cooperativity that stimulates additional interactions in signaling molecules [39].

### 7.2. Investigating the Role of UD in Src Signaling Using the optoSrc Approach

The essential roles of UD in the stabilization of Src oligomers in adhesive sites are utilized in optoSrc systems. Deletion of UD in optoSrc recruits the dimer to adhesive sites, suggesting that UD acts as a negative regulator of SH3 domain binding to PPP-containing proteins in adhesive sites [39], consistent with the role of UD in controlling the SH3 regulatory functions of Src activity [33]. Although the deletion of UD does not affect the residence time of optoSrc in adhesive sites, it slightly affects the optoSrc oligomer flux to adhesive sites, thereby increasing the percentage of cells forming invadosomes two-fold compared with cells expressing full-length optoSrc. Furthermore, increased enrichment of invadosome regulators (e.g., protein tyrosine phosphatase non-receptor type 6 (PTPN6)) by UD-deleted optoSrc is consistent with the stronger recruitment of UD-deleted optoSrc to adhesive sites [39]. On the other hand, UD deletion reveals the involvement of components of other signaling pathways, suggesting invasion control via regulators of membrane trafficking and functional coupling between regulators of actin-associated adhesion and extracellular matrix degradation in invadosomes [39]. These optogenetic systems may help to characterize the fine-tuning of UD in Src dimer-induced signaling pathways.

## 8. Protein Binding to UD

### 8.1. Effect of Calcium-Loaded Calmodulin (CaM) on the Intramolecular Interaction between UD and SH3

Addition of calcium-loaded CaM to USrc-induced remarkable CSPs and/or broadening for some residues (overlapping partially with ULBR) in UD. After the addition of CaM to USrcSH3, values of chemical shifts of SH3 returned to those of free SH3, indicating that the binding of CaM to UD competes with its intramolecular interaction with SH3 [36]. The CaM-USrcSH3 interaction reduced the capacity to bind to lipids, but the CaM-free SH3 interaction did not [36]. Suppression is mediated by UD, evoking the modulation of the UD-associated interaction by calcium levels through CaM.

Myristoylated alanine-rich C-kinase substrate (MARCKS) that is myristoylated, and IDR-containing proteins such as Src, are regulated by binding to CaM, inhibiting binding to plasma membranes and the sequestering of phosphatidyl 4,5-bisphosphate [75,76]. However, whether or not the UD of Src functions by a similar mechanism through CaM binding remains unclear.

Full-length Src is activated by both CaM and apoCaM in human cancer cells [77]. Fas-promoted survival signaling is mediated by CaM binding to the SH2 domain of Src [78], but whether or not CaM binding to UD affects the activity of full-length Src is unknown.

### 8.2. Regulation of N-Methyl-D-Aspartate Receptor (NMDAR) by NADH Dehydrogenase Subunit 2 (ND2) Binding to UD in Src

ND2, a subunit of complex I, acts as an adaptor protein anchoring Src to transmembrane protein NMDAR at post-synaptic densities [30,79,80]. ND2 binds to residues 40–49 within the UD of Src. ND2 does not interact with the SH3 or SH2 of Src, or with UD, SH2, or the SH3 of Fyn, despite being highly similar to Src. The UD of Src contributes to specific functions in synaptic NMDAR regulation by mediating specific protein–protein interactions [79]. The NMDAR/ND2/Src complex is disrupted by UD Src peptide (residues 40–49), thereby preventing Src from phosphorylating the cytoplasmic, intrinsically disordered C-terminal domain of the GluN2 subunit in NMDAR. NMDAR-related functions, long-term potentiation in CA1 hippocampal neurons [81], and inflammatory and neuropathic pain [82] are suppressed by the UD Src peptide, caused by downregulating NMDAR channel activity. It remains to be resolved how ND2 binding to the disordered UD of Src structurally affects the fuzzy complex of the Src N-terminal region, and vice versa, and likewise whether or not phosphotransferase activity is affected.

## 9. Phosphorylation of USrc in Src

Six different Ser/Thr phosphorylation sites (S12, S17, S51, T37, S69, and S75) have been described for mammalian USrc in vivo and in vitro (Figure 2A). This IDR of Src, which shares low sequence identity with other SFKs, interacts weakly with its targets through its limited and transient structural characteristics. It is thought that post-translational modifications additionally affect these interactions to confer the role of a protein regulation hub on IDR [41,83,84]. The FIMC composed of the SH4/UD scaffold with a structured SH3 in Src is conserved among SFKs. Specific phosphorylation events in the UD of Src enable the Src molecule to tune to a pleiotropic signaling hub, and to exhibit Src-specific regulatory functions, mediated by the FIMC apparatus conserved among SFKs.

Herein, the focus is mainly on S17 and S75 phosphorylation, which occur in human Src affecting its biological functions, since these phosphorylation sites are highly conserved in the Src proteins of phylogenetically distant species. Phosphorylation within the IDRs of other SFKs is described in a recent review article [32].

### 9.1. Characterization of Phosphorylated USrc by NMR: S17 and S75 Modulates USrc–Lipid Interactions

The unfolded region is the target of various kinases that modulate Src activity through phosphorylation. S17 is phosphorylated by protein kinase A (PKA), and T37 and S75 are phosphorylated by cyclin-dependent kinase 5 (Cdk5)/p25 (activator subunit) in vitro [35]. NMR directly reveals the structural effects of the phosphorylation of USrc in vitro through local chemical shifts, RDC perturbations, and PRE [33,35].

The phosphorylation of S17, near the boundary between SH4 and UD, disturbs a strongly positively charged region (preceding arginine residues 14–16) that is known to be important for interaction with negatively charged lipids, but this only results in very local conformational effects [35]. ΔPRE profiles of USrc and its phosphorylated forms were compared in [33]. S17 phosphorylation caused the PRE values to be closer to those predicted for a random coil for residues 15–20 and 35–45, pre-organized in FIMC, suggesting that S17 phosphorylation affects the compaction of the disordered region [33].

The phosphorylation of T37/S75 causes slight CSPs and similar RDC profiles outside the phosphorylation site compared with the unphosphorylated form, suggesting that phosphorylation does not contribute to local conformational changes, although this site is close to the partially structured ULBR. The origin of the observed biological effects of Src phosphorylation at residues 17, 37, and 75 may be mainly electrostatically mediated by phosphate-modulated interactions with other proteins and/or lipid membranes [35], and by affecting the FIMC directly, as shown for S17 [33].

Lipid-induced NMR CSPs have been compared for phosphorylated and unphosphorylated forms of USrc; S17 phosphorylation almost disappeared upon the interaction of SH4 residues with lipids, but the interaction of ULBR with lipids was only moderately affected [36]. By contrast, T37 and S75 phosphorylation diminished lipid interactions with ULBR, but exerted subtle effects on SH4 interactions with lipids. Phosphorylation electrostatically destabilizes the interaction of USrc with acidic lipids [36].

### 9.2. Real-Time NMR and Intact Cellular System Analyses of Crosstalk between Kinases and Phosphatases in USrc in Xenopus Egg Extracts

A site phosphorylated previously can inhibit or enhance phosphorylation at another site, and the order of phosphorylation eventually determines the final phosphorylation state [85]. Amata et al. [42] studied the phosphorylation of a USrc construct, a bacterially synthesized and post-translational modification-free construct, by time-resolved NNR in Xenopus oocytes and egg extracts, which are rich in kinases and phosphatases, to explore the complicated network of kinases/phosphatases based on the identified phosphorylation events. Three signals of chemical shifts indicating the phosphorylation of S75 (peak 1), S69 (peak 2), and S17 (peak 3) were observed in the egg extracts at different times and at different rates.

Phosphorylation at S17 is sensitive to PKA-related kinase broader-specificity inhibitor H-89 [42]. The addition of PKA increased S75 phosphorylation but not S69 phosphorylation. H-89 and PKA-specific inhibitor 6–22 inhibited phosphorylation at S75. The addition of H-89 at 30 min after preincubation of USrc in the egg extract also inhibited S75 phosphorylation, indicating that PKA-sensitive phosphatase activity abolishes CDK-mediated phosphorylation at S75. Inhibitor-1 of protein phosphatase 1 (PP1), which is activated by PKA, seems to be a candidate of the H-89 effector [42], although biochemical and mutational evidence is required to confirm this hypothesis.

S17 phosphorylation is not sensitive to PP1, because the addition of H-89 at 30 min after the preincubation of USrc in egg extracts did not affect this phosphorylation. However, roscovitine, an inhibitor specific for CDK-1, -2, and -5, inhibited S17 phosphorylation, implying that the activity of CDKs indirectly affects phosphorylation [42].

S69 phosphorylation was inhibited by roscovitine, similar to S75 phosphorylation [42], which seems to be catalyzed by CDK and related kinases, even though the amino acid sequence around S69 differs from the CDK consensus sequence (basic/polar-S/T-P-X-basic). Furthermore, the PKA dependency of S69 phosphorylation is different from that of S75 phosphorylation. This effect might indicate that S75 and S69 are targets of a different set of kinases and phosphatases, or it might suggest that phosphorylation of S69 cannot occur after S75 is phosphorylated [42].

Notably, real-time NMR spectroscopy enables the investigation of the regulation of phosphorylation/dephosphorylation, mediated by kinases and phosphatases, in IDRs of Src and other proteins in cells and cell extracts.

### 9.3. Stability Modulation by S75 Phosphorylation

The phosphorylation of S75 (S72 in chicken) is one of the mitosis-dependent phosphorylation sites associated with the mitotic activation of Src kinase activity in chicken fibroblasts [86,87,88]. By contrast, mitosis-independent phosphorylation at S75 occurs in Y79 retinoblastoma cells and some cancer cell lines, and is mediated by Cdk5/p35 kinase [89,90].

The pharmacological inhibition of Cdk5 kinase and Src S75A mutation both increased Src Y419 phosphorylation, and Src phosphorylated at Y419 was only phosphorylated at S75 [45]. The effects on SrcY419 phosphorylation are due to Cullin-5 function and to a reduction in ubiquitination of Src phosphorylated at Y419. Cdk5 inhibition and Src S75A mutation increase the stability of Src. Overall, S75 phosphorylation enhances Cullin-5-dependent ubiquitination [45].

Regarding the S75 promotion of degradation by the ubiquitin E3 ligase Cullin-5, this protein is a scaffold that engages ring box protein 2 (Rbx2, catalytic component), the Elongin BC complex, and suppressor of cytokine signaling (SOCS) box proteins. Members of this large adaptor protein family bind diverse substrates (ubiquitin target proteins) through their interacting domains [91]. Cullin-5 is necessary for the degradation of active but not inactive fibroblast Src [92]. The transformation of Cullin-5-deficient cells requires Src and other proteins including p130Cas, a substrate of Src. Cullin-5 suppresses transformation by binding tyrosine-phosphorylated Cas through the SH2 domain-containing SOCS6 adaptor protein. Src promotes Cas binding to SOCS6, but its silencing using a small interfering RNA (siRNA) had no effect on the steady state level of Src, although it did increase Cas protein stability, suggesting that Src is a target for other SOCS box proteins [93].

SOCS box proteins might include an adaptor for Src for the following reasons: (1) other SH2-containing SOCS proteins (1–5 and 7) apart from SOCS6 [94] bind to tyrosine-phosphorylated active Src; (2) phosphorylated S75 may be a phosphodegron because WD40 repeat and SOCS box-containing proteins (WSB1 and 2) are Cullin-5 adaptors [94], and WD40 domains can recognize the phosphodegron motifs (S/TP) that are activated by Cdk1 [95]; (3) Elongin A is a Cullin-5 adaptor, and its Elongin BC complex enables the large subunit of RNA polymerase II (Rpb1) to recruit the Cullin-5 ubiquitin ligase system by interacting with the phosphorylated S/P motif in the C-terminal domain of Rpb1 [94,96]. Although the actual adaptor has not been identified, phosphoserine 75 might enhance the availability of active Src to the Cullin-5 adaptor through regulation of the ULBR function as a controller of Src and lipid membrane proximity. Supporting this, the NMR analysis of USrc shows that phosphorylation electrostatically decreases the interaction between partially structured ULBR and lipid membranes [35,36]. Furthermore, because the chemical shifts of the hinge region (residues S75–G85) linking SH3 and UD are affected by PPP binding to SH3 (as shown by NMR analysis), the S75 phosphorylation-induced electrostatic effect of SH3-related interactions with other proteins or membranes cannot be ruled out. Cdk5 is responsible for S75 phosphorylation, and it has a PPP motif capable of binding to SH3 of Src [97].

### 9.4. Physiological Function and Src IDR Phosphorylation

#### 9.4.1. S17 Phosphorylation

Phosphoserine residues within the amino-terminus are increased following treatment with cAMP [98]. Ser17 is a consensus site for PKA phosphorylation [99], and Ser17 is phosphorylated in viral Src and cellular Src [100], but the physiological function remains obscure [3]. SrcS17 phosphorylation is induced by cAMP and nerve growth factor (NGF) to mediate the activation of Rap1 in PC12 cells, but the mechanism by which Ser17 phosphorylation directs Src activity is not clear [101]. However, growth factors stimulate Src translocation from the plasma membrane to the cytosol via S17 phosphorylation, interfering with the electrostatic interactions that mediate Src anchoring to lipid membranes [12,102], consistent with recent NMR results showing that S17 phosphorylation may regulate the interaction of SH4 with lipid membranes [36].

Despite great effort expended on researching the human tumor genome, the genetic alteration of Src has not been detected in human cancers. However, it remains possible that human oncogenesis is attributable to the deregulation of Src [103,104]. Comprehensive in vivo phosphoproteome analyses of an Hbx-transgenic mouse model of hepatocellular carcinoma (HCC) showed that S17 phosphorylation of Src is augmented, which is concomitant with the Y316 autophosphorylation of Lyn, another SFK member [105]. Shortening disease-free survival in patients with HCC is associated with high S17 phosphorylation levels. HCC cell line HepG2 stably overexpressing the non-phosphorylating mutant SrcS17A decreased the cell migration ability two-fold compared with cells overexpressing WT Src [105]. Phosphorylated Y530 that interacts with the SH2 domain, and autophosphorylated Y419 that regulates kinase activity, are not affected by S17 phosphorylation. However, interestingly, the S17A mutant suppressed Y256 phosphorylation of ROCK2 [105], which is involved in tumor invasion, but the detailed molecular mechanism by which S17 phosphorylation affects Src phosphotransferase activity and contributes to cell migration regulation has not been uncovered. The structural effects of S17 phosphorylation on the compactness and lipid membrane proximity of the FIMC might be related to the mechanism [33,36].

Ruiz-Saenz et al. [46] performed a phosphoproteomic analysis by mass spectroscopy using a full-length Src construct fused with C-terminal glutathione S-transferase (GST) expressed in breast cancer cell lines and human mammary epithelial cells, and the Src-GST protein was purified. Phosphorylation of S17 was decreased in more than 30 cancer cell lines, which affected Src UD-KD-mediated dimerization. The S17A mutant of Src increased the dimerization, suggesting that S17 phosphorylation is disruptive to dimerization. Mutagenesis of viral Src revealed that S17 phosphorylation is dispensable for transformation ability [99]. The antiproliferative role of Src is mediated by S17 phosphorylation via the activation of Src (Y419 phosphorylation) and Rap1 [106]. It is possible that Ser17 phosphorylation plays a suppressive role in tumorigenesis in some circumstances. Because the molecular process converting monomer to dimer and vice versa is difficult to study, this suppressive mechanism remains poorly understood. The dimer and monomer may differently modulate tyrosine-phosphorylating activity and/or exchange, thereby affecting molecular signaling activity and/or intracellular trafficking. A balance may exist between dimers and monomers, and this balance may be related to cancer biology. Recent progress on this novel regulation of Src via its IDRs may shed light on how Src functions in tumorigenesis and cancer pathology, including metastasis [107,108].

#### 9.4.2. S75 Phosphorylation

S75 phosphorylation affects the ULBR lipid-interacting function of UD [36] and regulates the stability of full-length Src [45]. How this phosphorylation affects the physiological functions of Src is an interesting question. In human lens epithelial cells, the Cdk5-dependent suppression of Src stability through S75 phosphorylation reduces central stress fiber formation by blocking the Rho-dependent phosphorylation of myosin [45,109]. S75 phosphorylation might regulate cell migration through the molecular switching of Rac-associated lamellipodia formation and Rho-associated stress fiber formation [45,109,110,111].

The use of optoSrc confirmed the role of UD in the stabilization of Src dimers/oligomers in adhesive sites, suggesting its ability to modulate Src behavior toward its substrates in vivo [39]. The UD-dependent regulation of the local density of optoSrc oligomers is sufficient to induce different sustained fluxes of the same optoSrc oligomers in adhesive sites [39], implying that this UD-mediated regulation of the half-life of Src might modulate incorporation into adhesive sites to dictate the formation of different actin-related structures. How S75 phosphorylation is linked to the fine-tuning of Src behavior related to the pleiotropic signaling hub remains to be determined.

Unlike phosphorylation/dephosphorylation, proteolysis can irreversibly terminate Src activity, leading to negative-feedback regulation that maintains the homeostasis of intracellular activity. S75-phosphorylated Src has been detected in some unsynchronized cancer cells with spherical morphology, but not in adherent and relatively flattened cancer cell lines [89]. Considering the proteolytic role of S75 phosphorylation, this may suggest a role in antagonizing Src activity in some Cdk5-expressing cancer cell lines [45]. During mitosis, Src activation is accompanied by SrcS75 phosphorylation in non-cancerous cells [88,112], implying that phosphorylation may control Src activity to prevent over-activation and thereby sustain normal cell conditions.

Post-mitotic CNS neurons have increased levels of the normal (fibroblast) form of Src and N-Src with higher specific kinase activity than the normal form [47,50]. Two forms of Src are expressed in undifferentiated and differentiated striatal neurons, but interestingly, one or two sites of serine phosphorylation in addition to S12 and S17 have been detected exclusively in the N-Src form in differentiated neurons [51]. This serine site is presumably S75 based on S75-identified phosphopeptide maps of unsynchronized Y79 retinoblastoma cells originating from CNS neurons [89]. Some neuron-related cancer cell lines contain both normal and N-Src forms, and unlike non-transformed CNS neurons, the normal form is also phosphorylated at S75 [89]. Even in post-mitotic CNS neurons, S75 phosphorylation might suppress activated N-Src to maintain the appropriate level of Src catalytic activity for normal CNS function.

Neurons have higher specific Src kinase activity and high levels of Cdk5 expression. The S75 phosphorylation function in CNS neurons is evident in mice mutated at S75. Phosphomimicking S75D [43] and non-phosphorylatable S75A [44] mutant mice exhibit specific roles of Src upon evading functional redundancy by SFKs, and possible regulatory roles of SrcS75 phosphorylation in physiological Src activities in post-mitotic neurons, such as age-related retinal ganglion cell loss [43] and the modulation of ethanol preference and consumption in the brain [44]. Perturbations in phosphorylation networks of major signaling proteins in specific tissues remain to be comprehensively explored.

The relevance of Src to neurodegenerative disease is becoming increasingly apparent. Dysregulated kinase networks in Alzheimer’s disease (AD) have been unraveled using proteomes and phosphoproteomes of temporal cortex tissue from patients with late-onset AD and age-matched, non-diseased controls [113]. Phosphorylation on SrcS75 and Cdk5Y15 was first reported for phosphosites related to the late-stage tau and oligodendrocyte pathologies of AD. The roles of CdkY15 phosphorylation remain obscure [114], although whether this phosphorylation promotes SrcS75 phosphorylation is critical in Cdk5/Src signaling.

In other biological systems, the adhesion G protein-coupled receptor (GPCR) latrophilin-2 (LPHN2) dictates the cardiac lineage commitment of Src and Cdk5, which are key downstream molecules of LPHN2 signaling [115]. LPHN2 activates P38 mitogen-activated protein kinase (MAPK) via the phosphorylation of SrcS75 and Cdk5Y15, but the relationship between Cdk5 and Src in these molecular signaling pathways remains unclear [115].

#### 9.4.3. Phosphorylation at Other Sites

Src is known to be phosphorylated at S12 by protein kinase C [116]. S12 phosphorylation is induced in fibroblasts by the treatment of activators of phosphatidylinositol turnover [117]. N-Src contains two additional sites of phosphorylation, one of which is Ser12, in addition to Ser17 in differentiated neurons [51]. However, the direct effects of S12 phosphorylation on Src kinase enzymatic activity are unclear. Phosphorylation might affect electrostatically positively charged and lipid membrane-interacting regions of SH4, which may promote translocation from the plasma membrane to the cytosol [76].

Glycogen synthase kinase 3β (GSK-3β) phosphorylates S51 in UD, concomitant with S493, leading to a decrease in Y419 phosphorylation in the possible desensitization process of Wnt3A signaling [118]. NMR-CPS was detected at this site during lipid and CaM binding by USrc [36] The effects of S51 phosphorylation on lipid binding by ULBR, IDR-assisted membrane trafficking, and dimer formation, remain to be addressed.

## 10. Concluding Remarks

**Figure 5:**
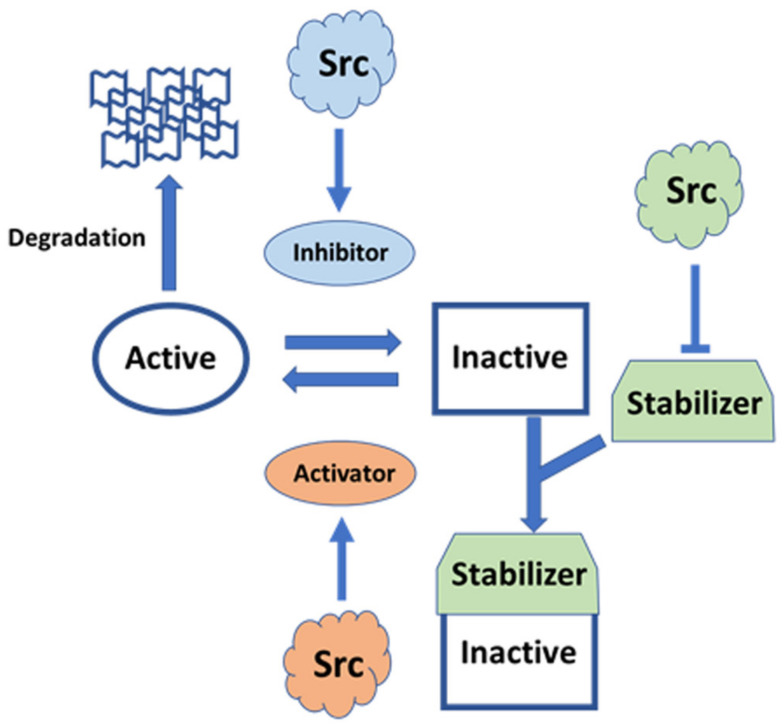
Pleiotropic signaling of Src involving Rho GTPase. Plasma membrane-anchored Rho GTPases are regulated by GTPase-activating protein (GAP) inhibitors and guanine nucleotide-exchange factor (GEF) activators. Activated Rho bound to GTP is inhibited by GAP inhibitors enhanced by Src [119,120]. Inactive Rho bound to GDP is activated by GEF activators promoted by Src [121]. GDP dissociation inhibitor (GDI) stabilizes the inactive state by inhibiting the exchange of GDP/GTP and membrane anchoring, which is inhibited by Src [122]. GDI possibly avoids Rho ubiquitin-mediated degradation.

Src functions in various tissues via cytoskeletal modulation, in both growing and post-mitotic cells. Src receives diverse signals from the external environment, and it transduces the translated signals intracellularly. The same Src molecule can receive multiple inputs and generate pleiotropic responses depending on temporospatial factors. In some cases, Src modulates the same effector, such as Rho, in opposite directions and in different ways (Figure 5).

**Figure 6:**
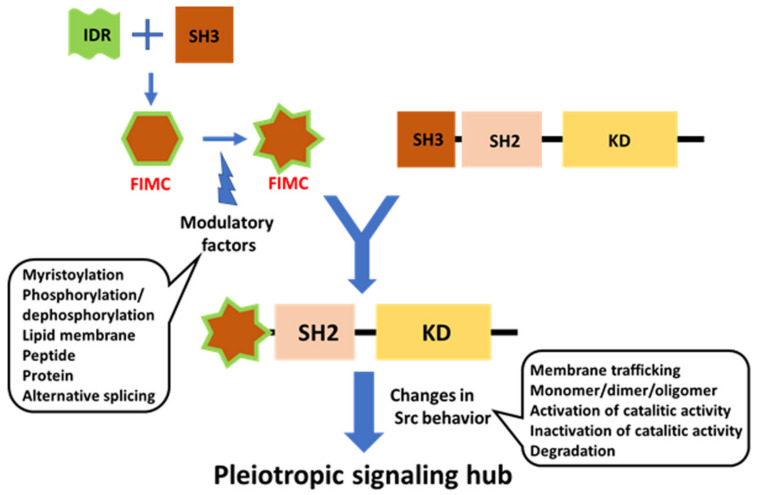
Schematic diagram of potential FIMC-directed Src regulation. The FIMC directs full-length Src behavior spatiotemporally. The FIMC comprised of IDR scaffolded by SH3 is modulated by post-translational modifications, such as myristoylation and phosphorylation; interactions with lipid membranes and proteins/peptides; and alternative splicing. The FIMC alters the behavior of the full-length Src molecule, including membrane trafficking, dimerization/oligomerization, switching of phosphotransferase activity, and degradation. Thus, Src functions as a pleiotropic signaling hub under spatiotemporal control.

Recent NMR analysis of the structure of the N-terminal IDR region has shed light on new regulatory roles of the IDR, which acts as a rope to anchor the modular structure to the lipid membrane (the classical model of Src regulation), consistent with recent optogenetic and chemical genetic approaches. The regulatory model has been integrated with the compact fuzzy structure formed with SH3 adjacent to the IDR, concomitant with the roles of phosphorylation in the IDR. Further integration with the canonical model, based on crystallographic analyses, in which SH3 acts as a switch for activation, will pave the way for the elucidation of all aspects of the modulatory mechanism of full-length Src activity. Individual SH4 and UD regions in SFKs function as sensors for diverse extracellular stimuli, using SH3 as a scaffolding domain to transmit these specific signals to the conserved core, composed of SH3, SH2, and KD, in various SFKs [34,56]. The FIMC that is formed on the SH3 scaffold exhibits plasticity at the molecular level to integrate complex signals with assistance through phosphorylation events within the IDR of SD4-UD. The phosphorylation of SD4-UD may affect the fuzzy complex structure directly or indirectly, by altering the interactions of the SD4-UD with lipid membranes and/or other regulatory proteins. SH3-SH2 regulatory domains may interact with KD in the active state in the absence of C-terminal phosphotyrosine 530, stabilized by a conformationally selective probe [123], and in major species of the Y530F mutant representing the active state [124], implying that modulated FIMC intervenes in canonical regulation through the conversion of open and closed conformations via SH3, a common component of the FIMC and the canonical regulatory complex [56]. The SH3 scaffold is a mediator that exchanges active and inactive states, as well as controlling direct sequestration by degradation, following information translated by the N-terminal FIMC such as membrane trafficking and monomer/dimer formation (Figure 6).

To fully elucidate the role of full-length Src as a temporospatial regulatory hub in pleiotropic signaling, novel NMR-based approaches will be needed. Further optogenetic and chemical genetic approaches will also be needed alongside new genetic animal models. High-speed atomic force microscopy linked with NMR is an effective tool for capturing the spatiotemporal dynamics of the whole IDR-containing protein [125,126,127,128].

Numerous reports concerning Src in cancer, neurodegeneration, and other pathological states have been published. However, although Src is a candidate therapeutic target, useful drugs are unlikely to be developed without elucidation of the structural basis of the regulation of Src function. The studies discussed herein, and future work on the structure and dynamics of full-length Src, will illuminate the multiple roles of this complicated regulatory system.

## Data Availability

Not applicable.
